# The National Magazine

**Published:** 1890-06

**Authors:** 


THE NATIONAL MAGAZINE

For April opens with an interesting article entitled "Chatterton; the Boy Poet," by Rev. Albert Danker, D. D., of the National University of Chicago; the "Current Value of Degrees" is by Dr. F. S. Thomas, M. D., Ph. D.; other articles are "The Columbus Society of Patriots of America," a laudable organization to cultivate patriotism in our American youth; "The Origin of the Name and Office of Justice of the Peace," by Rev. Joshua P. Bobb, LL. D.; and "Save Our Farmers," by F. W. Harkins, Chancellor of the National University, the non-resident or correspondence work of which rapidly developing institution is also explained in this number. Sample copy ten cents. Address, 147 Throop Street, Chicago, Ill.

